# Contrasting water, dry matter and air contents distinguish orthophylls, sclerophylls and succophylls (leaf succulents)

**DOI:** 10.1007/s00442-025-05686-4

**Published:** 2025-03-20

**Authors:** Byron B. Lamont, Heather C. Lamont

**Affiliations:** 1 Ecology Section, School of Molecular and Life Sciences, Curtin University, PO Box U1987, Perth, WA 6845 Australia; 2 27 Windchime Tce, Atwell, WA 6164 Australia

**Keywords:** Arid climate, Leaf density and thickness, Leaf succulents, Leaf texture, Leaf water content, SLA

## Abstract

Differences in leaf texture (hardness, thickness) distinguish orthophylls (soft leaves), sclerophylls (hard leaves) and (semi)succophylls (water-storing leaves). Texture is controlled by dry matter, water and air contents. Our aim was to a) identify the best index of succulence, b) assess how these three components vary with leaf type, and c) derive bounds for these properties among the four main leaf-texture classes. Eight contrasting species from the Namib Desert, South Africa were assessed for their leaf area (*A*), thickness (*z*), dry mass (*D*), saturated water content (*Q*), and relative volume of dry matter, water and air to derive various indices of leaf texture. *Q*/*A* (= *Q*_V_•*z*), where *Q*_V_ is saturated water storage per unit volume of leaf and *z* is leaf thickness is an ideal index of succulence. Specific leaf area (SLA) is more suitable as an index of hardness (SLA^−1^ = *D*/*A*) but only among non-succulents. Rising leaf specific gravity among sclero-orthophylls is due to replacement of air by dry matter but water among succophylls. Collation of 13 worldwide studies showed that orthophylls can be distinguished by a *Q*/*A* ≤ 0.45 mg water mm^−2^ leaf surface from succophylls with *Q/A* ≥ 0.9, such that there is a divergent relationship among plants regarding their water-storing properties. Semi-succophylls can be defined as having a *Q*/*A* > 0.45 to < 0.9, and sclerophylls can be separated from orthophylls by a SLA ≤ 10 mm^2^ mg^−1^ dry mass. The distribution of these leaf texture classes may vary greatly within, and especially between, local floras.

**Supplementary Information:**

The online version contains supplementary material available at 10.1007/s00442-025-05686-4.

## Introduction

As an adaptive response to their environment, texture is a fundamental morphological property of leaves. It is difficult to quantify and field ecologists often resort to a ‘feel’ test based on firmness to touch, brittleness when bent, and appearance of moisture when squeezed (Cowling and Campbell 1983, Edwards et al*.* 2000). But leaf texture must have a structural basis that can be expected to depend on the relative content and spatial arrangement of dry matter, water and air (Niinemets 1999; Pyankov et al. 1999; Roderick et al. 1999a; Read and Sanson 2003; Z Wang et al. 2022a, b). Whenever these components are quantified, they are shown to respond immediately (physiologically) and evolutionarily (genotypically) to differences in growing conditions (Groom and Lamont 1997; Fradera-Soler et al. 2021, H Wang et al. 2022a, b), justifying attempts to measure them. With protracted dry periods now occurring in the mid-latitudes due to climate change, there is increasing interest in the properties of sclerophylls and leaf succulents that enable them to cope better with drought than orthophylls (Lamont et al. 2015; Pérez-López et al. 2023; Yu et al. 2024).

Sclerophylls have been described as “hard, coriaceous and thick, breaking when folded” (Cowling and Campbell 1983). They are characterised by high leaf thickness and dry density (Lamont et al. 2015) that appears to be at the expense of water storage, which, however, is rarely measured. Orthophylls have been described as “leaves with ordinary texture, as opposed to sclerophylls” (Fosberg 1967) and “soft, thin and pliant when folded” (Cowling and Campbell 1983). Thus, they appear to contain moderate levels of dry matter, air and water. The term, mesophyll, is often used as a synonym, especially in the context of rainforest vegetation (e.g., Lamont et al. 2024) that we replace here as that is also a specific leaf size class used in the Raunkiaer system (Johnston and Lacey 1984).

We introduce a matching term to orthophyll and sclerophyll as applicable to leaf succulents, succophyll: “sucus” (Latin for juice/sap) + “phyll” (Greek for leaf), to avoid confusion with other succulent organs (swollen stems, bulbs, tubers; Pausas et al. 2018). Such leaves have a ‘grape-like’ texture releasing liquid on squashing (Cowling and Campbell 1983), attributable to special water-storing tissues, and appear low in dry matter and air (von Willert and Brinckman 1986; Skarpe 1996; Lamont and Lamont 2000), a topic to be examined here. In addition, Cowling and Campbell (1983) distinguished semi-succulents with a pliable feel as “fleshy…with a rubbery or gelatinous texture”. Malacophylls (malac = Latin for soft/pliable) is often used as a synonym for orthophylls, semi-succophylls and/or succophylls, essentially as the alternative class to sclerophylls (O’Leary 1994). Consistent with a recent definition that highlights their thinness and association with moist climates (H Wang et al. 2022a, b) and to avoid further confusion, we treat malacophylls as equivalent to orthophylls and use the latter term here instead.

Succophylls are present in all continents and are characteristic of the vegetation of the most seasonally hot and dry parts of Africa, the Americas and Eurasia in particular (Landrum 2002; Males 2017; Pérez-López et al. 2023). They have been identified in 80 families and are considered to account for ~ 13,000 species worldwide (Nyffeler and Eggli 2010). For example, the leaf-succulent family, Aizoaceae (syn. Mesembryanthemaceae), makes up 40% of the species-rich flora of the South African Western Cape (Milton et al. 1997), although this flora is usually described as sclerophyllous (Rundel et al. 2016). Some epiphytes, such as tropical orchids, possess high water contents and are included among succulents (Pérez-López et al. 2023) but might better be described as semi-succophylls (Gotsch et al. 2022). Thus, four leaf-texture classes can be recognized in plant communities that include leaf succulents: orthophylls, sclerophylls, semi-succophylls and succophylls, and their relative abundance is often used to characterize the vegetation (Cowling and Campbell 1983; Pate et al. 1984; Keith and Sanders 1990; Grubb et al. 2015; Gotsch et al. 2022).

Few community studies have included or recognized leaf succulents which implies bias and limits their ability to make widely applicable generalizations. Notable exceptions include Vendramini et al. (2002), who studied 13 nominated leaf succulents among 77 Argentinian species, and Grubb et al. (2015) who included 11 species that they considered leaf succulents/semi-succulents among 38 arid Spanish species. Neglect of succophylls is unfortunate as it is usually anticipated that generalities determined for sclero-orthophylls will not apply to leaf succulents (Roderick et al. 1999b). In concluding that dry-matter content of fresh leaves was a useful functional trait, Wilson et al. (1999) noted that it was not clear how relevant it would be for floras with many succulents. Vendramini et al. (2002) showed that relationships between leaf properties depended on whether or not succulents were included in the sample, while Grubb et al. (2015) noted that the physical properties of their succophylls were quite different from those of orthophylls.

Here, we examine, theoretically and empirically, how three standard measures of succulence relate to their underlying textural properties, such as leaf thickness (*z*), and dry matter and saturated water contents on total fresh mass (*D*_M_) and volume (*D*_V_) bases. See Table 1 for the meanings of symbols and acronyms. Our working hypothesis was that the four texture classes have a structural basis as outlined above. We were aware of the problem of comparing confounded variables (with one or more components in common) as they will be correlated by definition (Williams et al. 2022), unless they remain constant in a particular study. Thus we note, for example, that the type of relationship of *D*_V_ with SLA [= (*D*_V_•*z*)^−1^], proposed as of potential interest by Wilson et al. (1999), is already mathematically prescribed (it will be negatively curvilinear) so it is of little empirical value. If undertaken here, the aim was to determine which of the various components best explained the relationship statistically.

**Table 1:** Definitions of acronyms and symbols used in this study

Acronym, symbol	Definition
ρ_l_	Leaf specific gravity (*D*_V_ compared with mass of an equal volume of water)
ρ_Q+D_	Specific gravity on a mass basis (mass of dry matter and water at saturation compared with mass of an equal volume of water)
*A*	Leaf projected area
*D*	Dry matter mass per leaf
*D*_M_	Dry matter mass on a water-saturated leaf mass basis
*D*_V_	Dry matter mass on a leaf volume basis
*F*_a_	Fraction of leaf volume as air space
*F*_D_	Fraction of leaf volume as dry matter
*F*_Q_	Fraction of leaf volume as water
l	Referring to the leaf
LMA	The inverse of SLA (*D*/A = *D*_V_•z)
SLA	Specific leaf area (*A*/*D* = (*D*_V_•z)^−1^)
*M*	Saturated mass (*Q* + *D*) per leaf
*Q*	Saturated water mass per leaf
*Q*_M_	Water mass content on a water-saturated mass basis
*Q*_V_	Water mass content on a volume basis
(*Q* + *D*)/*D* = *Q*/*D* + 1	Index of succulence, total mass on a dry mass basis
(*Q* + *D*)/*A*	Index of succulence, total mass on a leaf area basis
*Q*/*A*	Index of succulence, saturated water mass on a leaf area basis
*V*	Volume per leaf
*V*_D_	Dry matter volume per leaf (dry density)
*z*	Leaf thickness (after Lamont et al. 2015)

See Table 2 for units used

Logically, mass-related indices are more likely to be related to mineral/organic-storage properties, and volume-related indices to water-storage properties. Because Roderick et al. (1999a) highlighted the need to recognize air as a leaf component, we examined how the relationship of the fraction of air on a volume basis (*F*_a_) varied with water (*F*_Q_) and dry matter (*F*_D_) that contributed to their specific gravity (ρ). This ground-breaking approach seems to have had no subsequent follow-up, possibly because determining the volume of dry matter and air is ‘too difficult’? We expected that increasing ρ of sclerophylls would be attributable to replacement of air by dry matter, and increasing ρ of succophylls of air by water. This analysis first required us to develop a technique to determine the volume of dry matter that might, in turn, be considered novel.

The volume (*V*) of a leaf is made up of the product of the projected area (*A*) and mean thickness (*z*) (*V* = *A* × *z*, Lamont et al. 2015). Contributors to leaf volume are dry matter, water and air. Leaf turgid mass (*M*) consists of its dry mass (*D*) plus saturated water content (*Q*) (*M* = *D* + *Q*). Dry mass and water content can be related to turgid mass on a fraction basis (*D*_M_ and *Q*_M_ respectively). They can also be related to turgid volume (*D*_V_ and *Q*_V_ respectively) that is equivalent to density (mass per unit volume). Specific leaf area (SLA) is given by *A*/*D* and corresponds to the inverse product of *D*_v_ and *z* (Witkowski and Lamont 1991). Increasing succulence can be expected to involve an increase in *Q*_v_ with a decrease in both the volume fraction (*F*) of air (*F*_a_) and dry matter (*F*_D_), and thicker leaves (*z*) imply greater succulence if it is due to water (and greater sclerophylly if it is due to dry matter). Three standard indices of leaf succulence have been used historically:

1. *Q*/*A* (Dilf, 1911; Cowling and Campbell 1983) = *Q*_V_*•z*;
2. (*Q* + *D*)/*D* (Lamont and Lamont 2000; Grubb et al. 2015) = (*Q*/*D* + 1) = (*Q*_V_/*D*_V_ + 1);
3. (*Q* + *D*)/*A* (Eccles et al. 2001) = *z*(*D*_V_ + *Q*_V_).

Note that *Q*/*D* may also be used as it just the second index minus the constant 1 (Ogburn and Edwards 2012; Ripley et al. 2013).

We undertook the supporting experimental work on eight species in the southern Namib Desert of South Africa, with four species fitting the leaf succulent class of Vendramini et al. (2002) based on their saturated water content (Lamont and Lamont 2000). The vegetation is part of the succulent karoo that Grubb et al. (2015) specifically noted would form a useful comparison with their study in semi-arid Spain. This comment probably arose as the authors were no doubt aware that this flora is considered the most succulent in the world (Cowling et al. 1998) and therefore we expected it to provide suitable benchmark species for comparison with those in other floras. Our aim was to determine for our eight species which of their components most affected these indices of leaf succulence, and which index best reflected the water-storage capacity of a leaf. More generally, we were interested in how the volumetric relationship between dry matter, water and air varied with changes in leaf specific gravity as a function of their leaf texture type. As another common index of leaf texture among orthophylls, comparisons were also made with SLA to see if it, or its components, *z* and *D*_V_, could be used as a surrogate for succulence.

Finally, by combining our data with all other data that included leaf succulents which we could locate, we tested the proposition that there is a universal relationship between water mass content per unit dry mass (*Q*/*D*) and SLA (= *A*/*D*) as concluded by Z Wang et al. (2022a, b) based on extensive data that, however, did not appear to include leaf succulents. The slope of this relationship gives *Q*/*A* (*D* cancels out) that we conclude here is the best index of succulence. It was not our aim to explore the adaptive advantages of the various texture classes in the context of particular habitat types (Landrum 2002) nor how various ecophysiological mechanisms are coordinated with the rest of the plant (H Wang et al. 2022a, b). This has already been examined in terms of ‘utilizable’ stored water, soil water availability and photosynthetic efficiency among succulent-karoo species (Flach and Eller 1994; Lamont and Lamont 2000; Ripley et al. 2013; Veste and Herppich 2021). Our central task was to describe the structural properties (dry matter, water, air) that distinguish the four leaf types and identify the most relevant variables that ecologists should measure in assigning species to them.

## Materials and methods

### Fieldwork

Leaves were collected from eight species growing wild at Groenriviersond, 500 km north of Cape Town, South Africa (30º 51' S, 17º 34' E). The species were selected to cover the full range of textures among perennials at the study site [SLA ranged from 2 to 20 mm^2^ mg^−1^, Lamont and Lamont 2000]. They were *Pteronia onobromoides* (Asteraceae, shrub to 50-cm tall, hard-leaved), *Salvia lanceolata* (Lamiaceae, shrub to 1 m tall, soft-leaved), *Eriocephalus africanus* (Asteraceae, shrub to 1-m tall, soft-leaved), *Stoeberia utilis* (Aizoaceae, syn. Mesembryanthemaceae, ground creeper, succulent), *Ruschia fugitans* (Aizoacae, syn. Mesembryanthemaceae, ground creeper, large-leaved succulent), *Zygophyllum morgsana* (Zygophyllaceae, shrub to 50 cm, semi-succulent), *Othonna cylindrica* (Asteraceae, shrub to 40 cm, succulent) and *Senecio* aff. *sarcoides* (Asteraceae, undershrub, small-leaved succulent). Nomenclature is as given in Eccles et al. (1999) and, from hereon, only the genus names are used. Leaves of all species were iso(bi)lateral and sessile (except *Salvia*). As this is a shrubland, all species were growing in the open so that differences in microclimate would have had no role in affecting the results. They varied from apparently sclerophyllous to highly succulent. On a water mass content per unit volume basis, *Q*_v_, 2 species were in the range 40 − 50%, 3 were 60 − 70%, and 3 were 80 − 95% (Lamont and Lamont 2000). Thus, the water-storing properties of the eight species studied formed a well-defined gradient that proved ideal for testing the hypotheses outlined here.

The study site lies in the southern portion of the Namib Desert. The vegetation is part of the succulent karoo and consists of clumps of climbers to woody shrubs up to 2-m tall (Eccles et al. 2001). The soil is red aeolian sand overlying an impenetrable silcrete hardpan at about 2-m depth. Rainfall was 79 mm in the year of the study although fog and dew are regular occurrences (Fradera-Soler et al. 2021).

### Laboratory work

Current season’s mature twigs (100–150-mm long) were removed from side branches of 6–8 plants of each species by cutting under water predawn. They were kept with their ends in water at 17.5–20.5ºC and covered with plastic bags for 1–4 days in the laboratory to promote full hydration. They were then recut under water and their pressure–volume relations determined following the protocol of Radford and Lamont (1992). The balancing pressure was achieved with a digital pressure chamber, model 1003, PMS Instruments, Corvallis, OR, USA. To obtain turgid (saturated) mass as needed for this study, wet weight values of twigs were extrapolated to Ψ = 0, i.e., full turgidity. Ten mature, full-sized leaves were removed from other stems, and these plus the original supporting twigs used were weighed, frozen at − 16ºC to rupture the cells and hasten drying, dried at 72ºC for 48 h and reweighed. From this, turgid mass of the twigs was used to obtain leaf turgid mass (60–95% of total mass for individual twigs).

Midpoint thickness of ten leaves from three plants was determined with callipers. Projected area (*A*) was obtained by placing 30 leaves or more diagonally on the conveyor belt of an area meter (Li-Cor 3000, Lincoln, NK, USA). Adjustments were made for the shape of leaves and their volume (*V*) determined geometrically (Lamont et al. 2015): five were cylindrical (*V* = π/4*z*•*A*) where *z* was diameter, two were laminate (*V* = *z*•*A* where *z* was thickness) and one was subulate (*V* = mean *z*•*A*), all lacking midribs. SLA [*A*/*D* = (*D*_V_•*z*)^−1^, where *D* = dry leaf mass and *D*_V_ = dry leaf density on a volume basis, Witkowski and Lamont 1991] was adjusted for leaf shape in the same way, and *D*_V_ and *Q*_V_ (dry matter and saturated water mass per unit leaf volume) were based on these measurements.

Volume of dry matter was determined by removing all, and only, mature leaves from 6 twigs, bulking and macerating after oven-drying as above to pass through a 1.1-mm mesh, then twice through a 0.3-mm mesh. The powder was then moistened with a wetting agent (1% Tween 20) in distilled water to form a thick paste. A cork borer (internal diameter 3.58 mm) was pushed into the paste, to produce an initial firm cylinder 20–40 mm in length. It was then placed on a paper tissue to absorb water over plastic sheeting on a fibrocement base. An iron rod of diameter 3.50 mm was pushed into the borer and tapped with a small hammer about 30 times, until water no longer squeezed out of the bottom. The pressure applied was up to 5.1 kg cm^−2^ but it was usually about 2.1 kg cm^−2^. The cylinder of compressed paste was forced out with the rod, and the ends cut with a razor blade as required to produce a perfect cylinder and its length and width determined with callipers. Three–five cylinders were obtained per species. They were dried at 65ºC for 40 h and kept in a desiccator until weighing.

### Properties assessed

Knowing *D*_v_ (*D*/*V*) and volume of dry matter per unit dry mass (*V*_D_/*D*), the contribution of dry matter volume — essentially cell walls, but including protein and most solutes — to total volume [(*V*_D_/*D*)(*D*/*V*) = *V*_D_/*V* = *F*_D_] and air, *F*_a_ = (1 – *F*_D_) were calculated. Thus, colloidal protein and other non-soluble substances were put with structure rather than cytoplasm or vacuole when estimating volume fractions (as in Roderick et al. 1999b). Some solutes may not have been adsorbed or held back by the cell-wall components during compression, but, even if some were lost, their contribution to volume would be negligible (< 0.01% of dry matter according to our estimates). Formulae for specific gravity (ρ) based on either non-air leaf volume (ρ_Q+D_) or total leaf volume (ρ_l_) (see Table 1) were as given in Roderick et al. (1999a).

Z Wang et al. (2022a, b) fitted a single relationship between SLA and *Q*/*D* for over 3,000 species distributed throughout most of the world’s vegetation types, especially in China and North America, that did not appear to include succophylls (confirmed in a later figure). We therefore decided to compare *Q*/*D* with SLA (*A*/*D*) (as did they among many other structural and ecophysiological properties of the species), whose slope produces *Q*/*A* — our preferred index of succulence, with all data sets which did include succulents that we could locate: ours and 12 others (Table 3). These were identified by feeding in the keywords: water content, succulent, SLA, into Google Scholar®. This yielded about 200 papers that were inspected to see if they provided data on *Q*/*M* and SLA, or they could be obtained from leaf dry matter (LDMC) or water content on a turgid mass basis. LDMC = *D*/(*D* + *Q*) that was inverted and 1 subtracted to give *Q*/*D. Q*/(*Q* + *D*) values were inverted, 1 subtracted, and re-inverted to give *Q*/*D*. Some data were obtained by measuring the length of bars in figures with callipers (to 0.05 mm) and converted to the indices of interest using the scale on the axis. Some data sets had to be rejected as their units were clearly incorrect and the solution was not obvious, while some listed papers proved impossible to obtain.

Half the papers used fresh mass to calculate *Q* that is misleading as it should be based on turgid mass if it is to be treated as a property of the plant rather than as a proximate response to the conditions of the day. No attempts to at least harvest the plants predawn were evident (see above for how turgid mass can be obtained, including extrapolation from pressure–volume curves). In this case, *Q*/*D* was multiplied by 1.1 (i.e., water content increased by 10%) to adjust for the error unless it was grown in hydroponics or water content exceeded 90% when any error would be small. For similar reasons, plants subjected to drought stress treatments were ignored. In passing, we note that many species had leaves with a *Q*_M_ of only 50% or less and wonder how successful the claims of turgid mass were.

### Leaf-type allocations

The 13 data sets were readily divisible into three groups: from the collated graph, only species considered to be succulents in previous papers were present up to a *Q*/*A* of 0.9-mg water per mm^2^ leaf surface; so we set this as the critical value to define succophylls. From 0.9 to 0.45 mg mm^−2^, about half the species were previously considered succulent and half non-succulent; we assigned these to the semi-succophyll class. Below 0.45 mg mm^−2^, only 2 of 134 species were previously considered succulent; so we assigned these to the orthophyll class. Within this class, SLA was < 10 mm^2^ mg^−1^ (LMA = 100 µg mm^−2^) for 91 species that seemed a reasonable and convenient, although somewhat arbitrary, boundary for defining sclerophylly as a subgroup within orthophylls (agreeing with Wright and Westoby 2002). [This also points to how LMA/SLA fails as an index of sclerophylly when succulents are included for most species with a *Q*/*A* > 0.9 mg water per mm^2^ leaf surface had a SLA < 10 mm^2^ mg^−1^ (i.e., implying that they are sclerophyllous when they are actually succophylls)]. These texture class bounds were applied to all species in the 13 studies and a summary table prepared.

### Statistics

Pairwise comparisons of attributes of interest were made using standard best-fit curves (linear, exponential, logarithmic, power) provided by the Cricket Graph III graphing program (Computer Associates, USA). As a directional relationship was expected from theory for all pairwise comparisons, a one-tailed test was used for *n* = 8, with *P* = 0.05 for *r* = 0.621, *P* = 0.01 for *r* = 0.700 and *P* = 0.001 for *r* = 0.788. Best-fit lines were only added when relationships were significant at *P* < 0.05. Normality/homogeneity of variance of the data were checked by determining residuals, Δ*Y*, for the best-fit lines and a) plotting against *X*, and b) ordering the residuals from smallest to largest and plotting against the order. If the data are normally distributed, when regressed a) will be uncorrelated (no pattern), and b) will be strongly linearly correlated (https://www.youtube.com/watch?v=-N9CIBYdsqY, Jiang Jingze PhD). None of the significant correlations did not conform to normality assumptions and the topic is not raised further under the Results.

Since the succulent, semi-succulent and sclerophyll-orthophyll classes showed quite different *Q*/*A* relations, we determined the best fit lines for each and added them to the overall graph. These were ‘forced’ to start from the origin, as clearly when SLA → 0, so must *Q*/*M* → 0. The data were not logged as a) this did not solve the quest for an approximate normal distribution, b) the fit was worse, c) the power function does not pass through the origin (0, 0) as required, d) the data did not follow a curvilinear path (i.e., no tendency to asymptote) as befits the power function, and e) logging relativizes the data so that there is no longer a linear quantitative relationship between the slopes that prevents them from being compared with each other (see comments in Williams et al. 2009).

## Results and discussion

### Indices of succulence

**Fig. 1:**
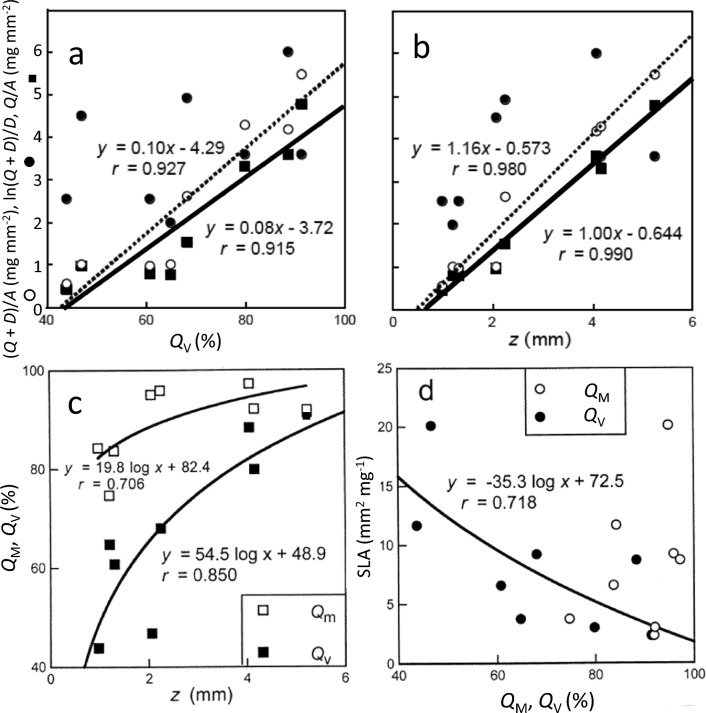
Three indices of succulence, ο (*Q* + *D*)/*A*, ● (*Q* + *D*)/*D*, ■ *Q*/*A*, plotted against a) saturated water content on a volume basis, *Q*_V_, and b) leaf thickness, *z*; c) saturated water content on a mass basis, *Q*_M_, and a volume basis, *Q*_V_, plotted against *z*; and d) specific leaf area, SLA, plotted against *Q*_M_ and *Q*_V_. Best-fit curves for (*Q* + *D*)/*A* given by dotted lines and *Q*/*A* by solid lines. *P* = 0.025% if *r* = 0.70, *P* = 0.008% if *r* = 0.85,* P* = 0.0005% if *r* = 0.92 (one-tailed). Note that ln[(*Q* + *D*)/*D*] is not correlated significantly with *Q*_v_ or *z*

Choice of index of succulence made a difference of up to four places in the rankings among our eight species. (*Q* + *D*)/*D* was uncorrelated with *Q*_V_ and *z* (Fig. 1a,b) and fails as an index of succulence as water storage is clearly at the expense of air not of dry matter, *D* (Roderick et al.*.* 1999c). Here, a *Q*_M_ of 75–97% can be accompanied by an air space of 2–51%, such that there is no relationship between *Q*_M_ and *Q*_V_ (not shown). Since succulence is a concept based on high water storage per unit leaf volume (*Q*_V_), and thicker leaves (*z*) with equal saturated water content are considered more succulent from a textural perspective, this makes *Q*/*A* (= *Q*_V_*•*z) the obvious choice as the *best index of succulence*. Interestingly, there was a clear logarithmic relationship between *Q*_V_ and *z* with *Q*_V_ gradually approaching 100% water content as leaves continued to thicken (Fig. 1c). A negative relationship was obtained by Roderick et al. (1999a,b) who proposed it as a general rule but it cannot apply to leaf succulents where *z* will increase as the cells swell with increasing water content (Fradera-Soler et. 2021). This finding needs more empirical research before its generality can be established — while it makes sense for succophylls it is unexpected for sclerophylls.

Compared with other species worldwide, some of our species showed exceptionally high levels of three common indices of succulence, leaf thickness (*z*) and saturated water content on a mass basis (*Q*_M_), but low levels of SLA and dry density on a saturated mass basis (*D*_M_), and an air-volume content (*F*_a_) that exceeded the upper values collated by Niinemets (1999) (Table 2). Adding our saturated water content on an area basis, *Q*/*A*, almost doubled the maximum previously recorded. The mean *Q*/*A* obtained from Z Wang et al. (2022a, b) for several thousand species is just 5% of the mean for our 8 species and highlights the profound effect that absence of species with water-storing leaves may have on data sets (see Fig. 3).

**Table 2:** Limits of physical properties of leaves worldwide compared with the eight species studied here

	Extreme records from the literature/Fig. 3			8 spp. in Namib Desert (this study)	
Attribute	Minimum	Maximum	Source	Minimum	Maximum
(*Q* + *D*)/*D* (g g^−1^)	1.75	14.30	Niinemets (1999)	4.0	36.5
Q/*D* (g g^−1^)	~ 0.1	> 26.0	Z. Wang et al. (2022a, b)	3.0	32.5
	0.5	32.5	Range from Fig. 3		
(*Q* + *D*)/*A* (mg mm^−2^)	0.30	3.22	Grubb et al. (2015)	0.55	5.50
*Q*/*A* (mg mm^−2^)	0.28	2.51	Grubb et al. (2015)	0.43 (m = 2.08) 4.80	
	0.01 (m = 0.10) 0.50		Z. Wang et al. (2022a, b)		
	0.02	12.96	Range from Fig. 3		
*F*_Q_ (mm^3^ mm^−3^ as %)	−	−		42.3	92.8
SLA = *A*/*D* (mm^2^ mg^−1^)	1.8 5.4 0.5	84.7 1270* 43.0	Niinemets (1999) Z. Wang et al. (2022a, b) Range from Fig. 3	2.3	20.1
*D*_V_ (μg mm^−3^)	92	1330	Niinemets (1999)	24	222
*D*_M_ (g g^−1^ as %)	7.0	37.0	Grubb et al. (2015)	2.6	25.4
*z* (mm)	0.06	1.96	Niinemets (1999)	0.97	5.23
*F*_a_ (air volume/leaf volume)	0.10 0.01	0.36 0.57	Niinemets (1999) Roderick et al. (1999a)	0.02	0.51

The first four attributes are common indices of leaf water-storage capacity. The fourth index (*Q*/*A)* is considered the best index here. m = mean

*needs checking

There were strong positive linear correlations between both saturated water content on a volume basis (*Q*_V_) and *z* for two of the indices of succulence, (*Q* + *D*)/*A* and *Q*/*A*, but no linear/curvilinear fits with the mass-based index, (*Q* + *D*)/*D* (Fig. 1a, b). (*Q* + *D*)/*A* is equivalent to (*Q*_V_*∙z* + *D*_V_*∙z*) and *Q*/*A* equals *Q*_V_*∙z* so that their significant relationships with *Q*_V_ and *z* is no surprise as they will be correlated by definition (see Williams et al. 2022 on ‘spurious correlations’). Garnier and Laurent (1994) reported a positive slope of 0.5 between (*Q* + *D*)/*A* (= *Q*_V_/*D*_V_ + 1) and *z* for grasses whereas ours was 1.2, no doubt due the additive effect of increasing water content with rising succulence. Their *z* values stopped 750 μm before ours started while ours continued until 5230 μm. Roderick et al. (1999b) also obtained a positive relationship between *Q*/*A* and *z* for non-succulents (even though their *z* values stopped before ours started) but their slope was lower (0.4 vs 1.0) for the same reason.

### Water *vs* air

**Fig. 2:**
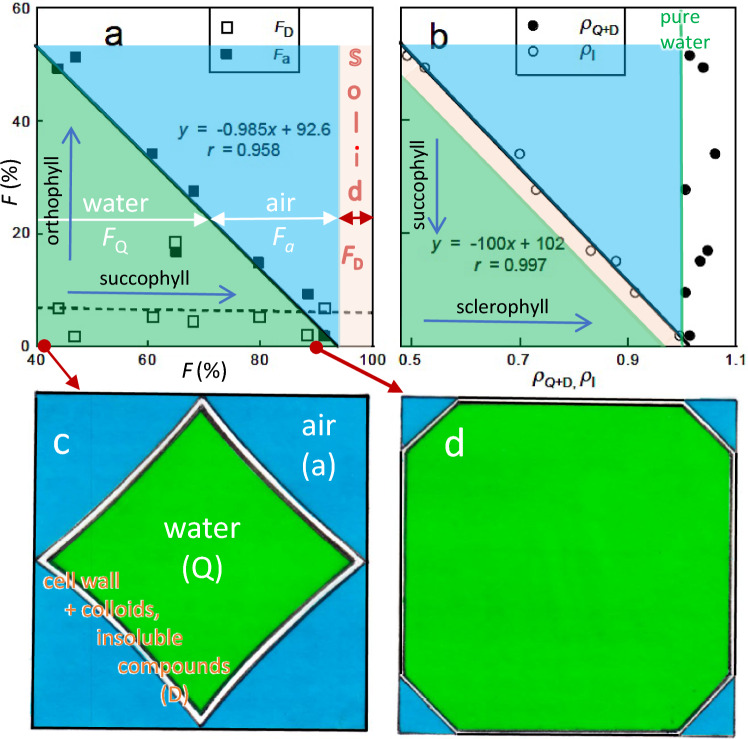
Volume fractions of major cell components. a) *F*_D_ (fraction of dry matter, orange area) and *F*_a_ (fraction of air, blue) plotted against *F*_Q_ (fraction of water, green); b) (*F*_Q_ + *F*_D_) plotted against specific gravity on a leaf mass basis, ρ_Q+D_, and leaf volume basis, ρ_l_ (note ρ_Q+D_ is slightly denser than pure water); c) Model of relative volume of cell components from data in Fig. 1, 2a, b and unpublished (protein); left cell based on *F*_Q_ = 40% (≡ *Salvia*), right on *F*_Q_ = 90% (≡ *Ruschia*). Green = *Q*_v_, blue = *F*_a_, white = cell wall (*F*_M_ – *F*_protein_), inner line = *F*_protein_. No attempt has been made to represent true cell shapes only to represent the relative size of the components accurately. Because of the colloidal properties of protoplasm its volume cannot be defined, so it is separated out on the inner wall as *F*_protein_, and all water is treated as in the vacuole. Solutes, of negligible volume, are included in *F*_D_ but would be dissolved in the vacuole. “Solid” is a euphemism for “dry matter”. For ease of comparison, model assumes an average cell of both occupies the same space; in practice, the more succulent species has (much) larger cells (e.g., Codignola et al. 1990)

As the fraction of water, *F*_Q_, increased from 42 to 93%, the fraction of air space, *F*_a_, decreased from 51 to 2% in a strongly linear manner, while the fraction of dry matter volume,* F*_D_, varied little at ~ 6.4% (Fig. 2a). Thus, saturated water content on a volume basis increases at the expense of air not of dry matter. This means for an increase in *Q*_V_ from 40 to 90% water, cell wall area increases by only 33% so that cell wall thickness is reduced by 25% for a constant dry matter volume (Fig. 2c).

Overall, succophylls have relatively large, thin-walled cells, a protoplast engorged with water, and tiny intercellular air spaces that would limit diffusion in the apoplast. This is illustrated well in the anatomical work of Codignola et al. (1990) and Fradera-Soler et al. (2021). Thus, Roderick et al. (1999c) correctly emphasize the need to recognize air (*F*_a_) as the third fundamental property of leaves (although it is rarely measured). Since the contribution of air to leaf volume varied by 50% in our species, *Q*_M_ and *Q*_V_ were completely uncorrelated. This means that using *Q*_M_ or *D*_M_ as a surrogate for *Q*_V_ or *D*_V_, as undertaken in some functional ecology studies (Weiher et al. 1999, Z Wang et al. 2022a, b), could be misleading. Thus, unlike Wilson (1999), we cannot advocate substituting *Q*_M_ for *Q*_V_ on theoretical grounds as they are not structurally equivalent (the presence of air is ignored in *M*). On practical grounds, the only additional measurement needed to estimate leaf volume (*V*) is *z*, which appears to be an even more fundamental functional property than both *M* and *V* (see below).

Specific gravity for *Q* + *D* (ρ_Q+D_, i.e., omitting air spaces) varied from 1.007 to 1.034 with a mean of 1.025 ± 0.020 (SD) and showed no relationship with *F*_a_ (Fig. 2b). Specific gravity for the whole leaf (ρ_l_, i.e., including air spaces) ranged 0.492–0.995 with a mean of 0.763 ± 0.184 and had an almost perfect linear relationship with *F*_a_. Our ρ_Q+D_ and ρ_l_ values are in the same range as those for non-succulents (Roderick et al. 1999a, b) so that there is nothing special about overall ρ values among succulents. But ρ_Q+D_ showed no relationship with fractional air space (*F*_a_) that is excluded from its formula (positive for Roderick et al. 1999a) and ρ_l_ showed a strong negative relationship with *F*_a_ (positive for Roderick et al. 1999a). This points to a basic difference between the structure of sclero-orthophylls and succophylls: ρ_l_ rises through an increase in dry matter density (*D*_V_) accompanied by a decrease in water content (*Q*_V_) among non-succulents, whereas ρ_l_ rises through an increase in water at the expense of air among succulents. Volume of dry matter (*F*_D_) played no role in interpreting the wide variation in *F*_a_ and *Q*_V_ among our species as it varied little (Fig. 2b).

### Relationships with SLA

There was no relationship between the three indices of succulence and SLA (Fig. A1 in Supporting Information). However, SLA declined logarithmically with increase in *Q*_V_, a component of *Q*/*A* (Fig. 1d). *Q*_V_ (and to a lesser extent *Q*_M_) increased at a decreasing rate as *z* increased (Fig. 1c). The lack of a (negative) correlation between *Q*/*A* (= *Q*_V_*∙z*) and SLA [= (*D*_V_*∙*z)^−1^] was unexpected because of their common, but inversely related, *z*. This must be due to the counteractive, erratic relationship between *Q*_V_ and *D*_V_ (Fig. A2). Note that *z* also increases as sclerophylly (the opposing textural type to succophylly) increases (Skarpe (1996; Lamont et al. 2015). This means that leaf mass area (LMA), the inverse of SLA, will fail as an index of sclerophylly (Groom and Lamont 1999) when succophylls are included in the study as both are characterized by high *z* even though *D*_V_ may stay low*.* But LMA should still serve as an index of drought tolerance among both leaf types where it is due to high *z* values (Lamont and Lamont 2000; Lamont et al. 2002, 2015).

**Fig. 3:**
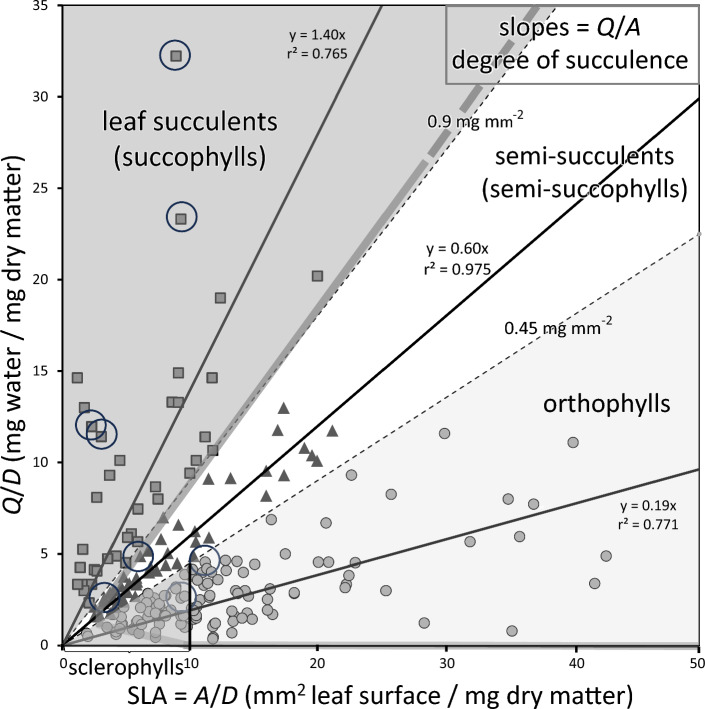
Relationship between saturated water content on a total leaf mass basis, *Q*/*M*, and SLA among 277 species from this study (data ringed) and 12 other studies (summarized in Table 3) that included succulent-leaved species and possessed *Q*/*D* and SLA data (or could be derived from their data) whose net slope yields our preferred index of succulence, *Q*/*A.* Four groups of species can be recognized: 1) high *Q*/*A*, representing leaf succulents (succophylls, grey squares), 2) low *Q*/*A*, representing sclerophylls (grey circles in triangle to left of SLA = 10 mm^2^ mg^−1^) and orthophylls (grey circles to the right of SLA = 10 mm^2^ mg^−1^), and 3) an intermediate group of semi-succophylls (black triangles). The boundary slopes between texture types are based on previous allocations in the literature with all of the upper class considered succulent, and half of the intermediate class considered succulent and half considered non-succulent. All line fits are significant at *P* < 0.001. The broad grey lines represent the outer bounds of the data in Z Wang et al. (2022a, b) showing the absence of succophylls in the data set and thus giving them grounds for fitting a single curve

Collation of SLA and *Q*/*D* data for 277 species, which included our data plus 113 species that fitted the succulent or semi-succulent classes, showed a broad scatter of points with crowding towards the base of both axes and two ‘arms’ spreading semi-vertically and semi-horizontally from the origin (Fig. 3). There is clearly no overall relationship between SLA and *Q*/*D*. The succophyll group was readily identified as representing the upper ‘arm’, with its Q/*A* (= *Q*_V_.*z*) ≥ 0.9-mg water per mm^2^ leaf surface and SLA under 15 mm^2^ mg^−1^ (see Methods). The overall slope of 1.40 mg per mm^2^ accounted for 77% of variance and attested to the high *Q*_V_ (high water, but minimal air and dry-matter, contents) and leaf thickness (*z*) of succophylls.

**Table 3:** Leaf texture types recognized in this study as defined by their saturated water content (*Q*/*A*, degree of succulence) and SLA

Leaf texture type	Saturated water content (*Q*/*A*)	SLA
Succophyll	≥ 0.9 mg water/mm^2^ leaf surface	Not restricted (usually < 15 mm^2^ mg^−1^)
Orthophyll	≤ 0.45 mg water/mm^2^ leaf surface	≥ 10 mm^2^ leaf surface/mg dry mass
Sclerophyll	≤ 0.45 mg water/mm^2^ leaf surface	< 10 mm^2^ leaf surface/mg dry mass
Semi-succophyll	> 0.45 − < 0.9 mg water/mm^2^ surface	Not restricted (usually < 25 mm^2^ mg^−1^)

By contrast, the lower ‘arm’ comprised the orthophyll group with a Q/*A* ≤ 0.45 mg mm^−2^ and SLA up to 45 mm^2^ mg^−1^. The overall slope of 0.19 mg/mm^2^, accounted for 77% of variance and represented just 14% of the volume of water per unit leaf area as possessed by the succulents. This attests to both the moderately high air content (Fig. 2) and thinness of orthophylls. Nevertheless, some orthophylls with high SLA can have high water contents (> 90%, Q/D > 10). The high dry density (*D*_V_) subgroup with SLA (= (D_V_.z)^−1^) < 10 mm^2^/mg is designated here as sclerophyllous but did include two species that were originally described as succulent in the original papers. These exceptions lend support to using the more rigorous criterion of Q/*A* rather than subjective impressions, if identification of succulents is one of the objectives of the study. There was also a substantial intermediate group, with a Q/*A* of 0.45 − 0.9 mg mm^−2^ and SLA < 25 mm^2^ mg^−1^ in this data set, that we recognize here as semi-succophylls. Their slope (water content), that accounted for 98% of variance, was three times that of the orthophyll class and less than half that of the succophylls. The criteria summarizing each of the four texture types are given in Table 3.

### Overall trends

The overall pattern in Fig. 3 contrasts with that of Z Wang et al. (2022a, b) who fitted a single curve to their data set that possessed many species with a *Q*/*M* > 5 but few with a SLA < 5 mm mg^−1^ that are the hallmark of leaf succulents. That is, the succulent ‘arm’ was omitted (although we reversed the axes, treating SLA as the independent variable). This means that the quest for a universal water-storing curve (‘physics envy’ *sensu* Lamont et al. 2023) will be unrewarding and, as a hypothesis, does not do justice to the rich structural variation in leaf texture that actually exists in nature. When the data cover the full range of textures, they readily fall into two major groups that are bidirectional: one stores water at the expense of air as SLA increases, and the other stores dry matter at the expense of air as SLA decreases. Thus, SLA can never serve as an index of the water-storing properties of leaves for it takes neither water nor air into account (Fig. 2). This is highlighted well by our own data ringed in Fig. 3.

Allocation of species to the texture classes recognized here shows that all four were well represented which will have served to minimize bias in the data set (Table 4). Interestingly, of the eight flora studies, all but one possessed species in all categories — the exception was the Italian alpine study that (intentionally) lacked sclerophylls. Even saline marshlands and grasslands may contain a few sclerophylls. This implies that there is a myriad of solutions, other than leaf texture, to dealing with drought or salinity, for all were conducted in arid or semi-arid environments, including roof tops. The semi-arid Spanish flora was notable for its abundance of semi-succulents, and the Argentinian woodland for the abundance of all four classes. Mistletoes (and parasitic plants generally) are usually considered to be semi-succulents and their eucalypt hosts to be sclerophylls that was confirmed here. The so-called succulent epiphytes were more correctly only semi-succulent, a concept not used in any of the papers reviewed here. Halophytes are usually considered to be semi-succulent, although several could be categorized as true succulents using our criteria, or the saline growth conditions converted them to true succulence.

**Table 4:** Summary of 13 studies that included ecophysiology of succulent or semi-succulent species, with numbers corresponding to number of species assigned to one of four texture classes as defined here

Context of study	Orthophyll*	Semi-succophyll	Succophyll	Sclerophyll	Reference	Notes
Semi-arid shrubland, Spain	4	15	4	5	Grubb et al. 2015	Semi-succulent species dominated the flora
Succulent karoo, Namib Desert, South Africa	1	2 C3	2 C3 + 2 CAM	1	This paper (see Fig. 3)	The most succulent flora assessed so far – water loss an inverse function of succulence; CAM spp are the most succulent
Semi-arid woodland, Argentina	27	11	15	29	Vendramini et al. 2002	Greatest texture range of all studies
Roof top candidates from Mediterranean-climate regions, grown in E Australia #	5	1	2	3	Guo et al. 2021	Survival not a function of succulence
Mistletoes and eucalypt hosts, south-eastern Australia #	0	All 4 mistletoes, some eucalypts	0	Most of 17 eucalypts	Richards et al. 2021	Only extreme or mean values assessed here as raw data not available
17 ‘succulent’ and 29 ‘non-succulent’ epiphytes, cloud forest, Cost Rica #	0	17	0	29	Gotsch et al. 2022	Only means available
Halophyte, *Crambe maritima*, under various NaCl concentrations, including control	0	0	1 (all treatments)	0	de Vos et al. 2010	Succulence increased with NaCl concentration
Saline marshland, cool desert, NW China	5	3	6	3	Wang et al. 2015	Halophytes semi-succulent and succulent
9 species of *Clusia* varying in photosynthetic pathway, cultivated England	0	3 CAM/C3 + 3 C3	3 (CAM)	0	Barrera Zambrano et al. 2014	CAM species most succulent
Roof top candidates from ‘harsh habitats’, grown in cold maritime Halifax, Canada #	12	1	1	6	Lundholm et al. 2015	*Plantago maritima*, not *Sedum*, most succulent
Steppe grasslands, Hungary	14	2	1	4	Krasser and Kalapos 2000	Even grasslands may contain succulents
Succulent alpine plants, Italy #	1	4	1	0	Codigdola et al. 1987, 1990	Swollen parenchyma cells illustrated, leaf area corrected to one side rather than two
Eight populations of desert halophyte, *Lycium ruthenicum*, in NW China #	0	3	5 (mean)	0	Li et al. 2021	SLA values incorrect (multiplied by 10^4^ to give cm^2^ g^−1^ as used in rest of this table)
Total	70	75	43	91	= 277	

*Excludes sclerophylls, #Fresh mass corrected to saturated mass as required (see Methods)

As expected, our eight species reached among the highest levels of succulence, water content and leaf thickness recorded among previous collations of non-succulent floras (Table 2, Fig. 3). But Table 4 makes it clear that succophylls and semi-succophylls are well-represented in arid and semi-arid floras world-wide. And Fig. 3 shows that the *Q*/*A* of two other species exceeds that of the highest in the Namib data set (12.96 *vs* 4.80), so that any suggestion that ours are ‘outliers’ cannot be supported. In addition, while our *Ruschia fugitans* has a mean *z* of 5.2 mm, *F*_a_ of 2.5% and *Q*_v_ of 93%, Fradera-Soler et al. (2021) studied two *Crassula* species from South Africa with similar trait values. *Othonna opima* is native to the southern Namib Desert, as with our *Othonna* species, with mean *z* of 9 mm (Flach and Eller 1994). Inspection of many other Aizoaceae and Crassulaceae species shows that leaves of these thicknesses are not uncommon. *Q*_V_ of two of the cultivated Aizoaceae species in South Africa assessed by Ripley et al. (2013) also exceeded 93%.

### Other comparisons with our data

There has been some interest in using *D*_M_ as a surrogate for *D*_V_ (as it is easier to measure) or as a functional attribute in its own right (Wilson et al. 1999). They were closely correlated in our study (not shown), as in Garnier and Laurent (1994) and Niinemets (1999). This explains why Vile et al. (2005) were able to replace *D*_V_ by *D*_M_ in the formula SLA = *A*/*D* = (*D*_V_*∙z*)^−1^ to estimate leaf thickness, *z*, as *D*_M_ is correlated with *D*_V_. This requires the extra step of measuring fresh mass (ideally turgid mass) if this was not otherwise of interest. Its only advantage might be that it gives average *z* rather than measuring *z* directly as it is usually only taken at the midpoint, or is overestimated among terete leaves (Lamont et al. 2015), that might introduce errors in the value of *z* if any problem is not recognized.

There has also been interest in the relationship of *Q*_V_ and *Q*_M_ with SLA in the hope that water content need not be measured. Among the two components of SLA, *D*_V_ was uncorrelated with *Q*_V_ (Fig. A2 in Supporting Information) whereas *z* was strongly correlated logarithmically with *Q*_V_ (Fig. 1c). The net effect was a weak negative curvilinear relationship between SLA and *Q*_v_ (Fig. 1d), clearly controlled by *z*, a relationship opposite of that obtained by Roderick et al. (1999a, c) for orthophylls. However, there was no relationship between SLA and *Q*_M_ (Fig. 1d) whereas Stewart et al. (1990) and Shipley (1995) observed positive trends for non-succulents. Vendramini et al. (2002) also observed no relationship between SLA and *Q*_M_ for Argentinian stem/leaf succulents. This is consistent with the large data set in Fig. 3, as the plotted *Q*/*D* is a function of *Q*_M_ via [(*Q*/*D*)^−1^ + 1]^−1^. Garnier and Laurent (1994) observed a negative relationship between *D*_V_ and *Q*_M_ (essentially a trade-off between dry matter and water, as expected by Shipley 1995), as did we (not shown) but our slope was much steeper (0.114 vs 0.075).

### Special features and limitations of this study

In these days of ‘armchair ecology’ (Pausas et al 2024), it is rare for plant species of special interest in remote locations to be studied in situ (the current study involved several visits to the study site 500 km north of Cape Town) — see Veste and Herppich (2021) for an exception. Most ecomorphology/physiology studies on leaf succulents are done on well-watered and fertilized plants in greenhouses (Ripley et al 2013; Fradera-Soler et al. 2021). This can be expected to have a distorting effect on the data, especially among CAM species, as included here (Lamont and Lamont 2000), whose photosynthetic type is environmentally induced (Ripley et al. 2013).

While we achieved the goal of natural growing conditions here, it was only possible for a team of two to study eight species simultaneously, so that their growing conditions were identical (Veste and Herppich 2021 only managed two, and Fradera-Soler et al. 2021, five). This limitation was addressed by a careful choice of species to cover as far as possible the full range of leaf-texture types present in succulent karoo rather than necessarily be representative of the flora. Our results fully justified this approach. Nevertheless, it provided a small sample for each leaf type that was largely overcome by adding results from 12 other studies that included succophylls (43) with the largest category (sclerophylls) represented by 91 species. Inspection of Fig. 3 and comparison with the equivalent figure in Z Wang et al. (2022a, b) shows that the data we used would benefit from species with higher SLA in the non-sclerophyll categories, perhaps by combining with the latter? The SLA values reached in Z Wang et al. (2022a, b) are an order of magnitude greater than the highest recorded by Niimetes (1999) (Table 2) that might even benefit from a re-appraisal?

We note our finding that *Q*_V_ and *z* are correlated (Fig. 1c) disagrees with that of Roderick et al. (1999a, b), who did not include succulents, and there is a case for more research on this topic. Perhaps the most novel part of our paper is the emphasis, and defense of, the volumetric, rather than mass, dimensions of leaf structural traits. The range of values we used is exceptionally wide and the line fits almost perfect so further assessments would have negligible effect on the pattern. The only reservation is the unexpected constancy of dry matter volume that needs to be checked on other species, although this aspect of leaf structure has been ignored since the theoretical work of Roderick (1999).

## Conclusions

Quantitative analysis and comparisons among 13 data sets that were considered by their authors to include leaf succulents (here termed succophylls) shows that the other three leaf textures, orthophylls, sclerophylls and semi-succophylls, are also readily distinguished. For a given projected leaf area, *A*, water-storage capacity is dependent on the fraction of volume occupied by water (*Q*_V_) and leaf thickness (*z*). This means that *Q*/*A* (= *Q*_V_•*z*) is an ideal index of succulence. The relationship between SLA and its components (*D*_V_, z) and other structural attributes (*Q*_V_, *Q*_M_) assessed here sometimes had parallels with values in other studies of non-succulent species. The strong positive relationship between *Q*_V_ and *z* has not been noted before: here, the thicker the leaf, the higher its water content (the opposite of sclerophylls) — this neglect may be because *z* is often replaced by the volume–surface ratio, that yields *z* when the constants are removed. Measures of specific gravity (ρ_Q+D_ and ρ_l_) have similar values to those for sclero-orthophylls but they have a different relationship with the air space (*F*_a_), highlighting differences in control of ρ_l_ between the two main texture classes.

When our data were combined with 12 other studies that included leaf succulents, it is clear that, when *Q*/*D* and SLA (*A*/*D*) are compared, the slope, *Q*/*A*, among succulents (succophylls) and sclero-orthophylls is completely different. This means that a universal curve between *Q*/*D* and SLA is neither possible nor desirable, despite recent support (Z Wang et al. 2022a, b). SLA fails as a potential index of succulence as water and air contents are not taken into account. High leaf thickness, *z*, holds the key to the special physiological and structural features of succophylls. Since succulence is a structural concept, *Q*_V_ (water content on a volume basis) is a much more useful parameter than *Q*_M_ (water content on a mass basis) in understanding its relationship with other variables.

Despite the small sample size, careful choice of species and habitat in our study not only produced values of many parameters that exceeded those previously collected (Table 1) but highlighted the great range in leaf textures that may exist in, as well as between, various floras (Fig. 3, Table 3). Care needs to be taken to distinguish the four leaf-texture types in future studies as we show that their ecomorphology/physiology properties are quite distinctive. While only < 5% of the world’s flora comprises leaf succulents, they have adaptive features of increasing interest under the expanding drought, heat and fire-prone conditions associated with climate change in the mid-latitudes, and special efforts should be made to include them in future ecomorphological/physiology studies (Pérez-López et al. 2023). The challenge now is to test the robustness of these proposed bounds to the four texture classes and see how their contribution to their floras compares with the assignments reported here.

## Supplementary Information

Below is the link to the electronic supplementary material.

Supplementary file1 (PPTX 79 KB)

## Data Availability

Data are available as a Dryad file, 10.5061/dryad.ksn02v7g5.
