# Supplementary material for: Contrasting water, dry matter and air contents distinguish orthophylls, sclerophylls and succophylls (leaf succulents)
Source: Oecologia. 2025 Mar 20;207(4):54. doi: 10.1007/s00442-025-05686-4 (PMC11926024; doi:10.1007/s00442-025-05686-4)
Supplement: Supplementary file 1 — Supplementary file1 (PPTX 79 KB) [file 442_2025_5686_MOESM1_ESM.pptx]

## Slide 1
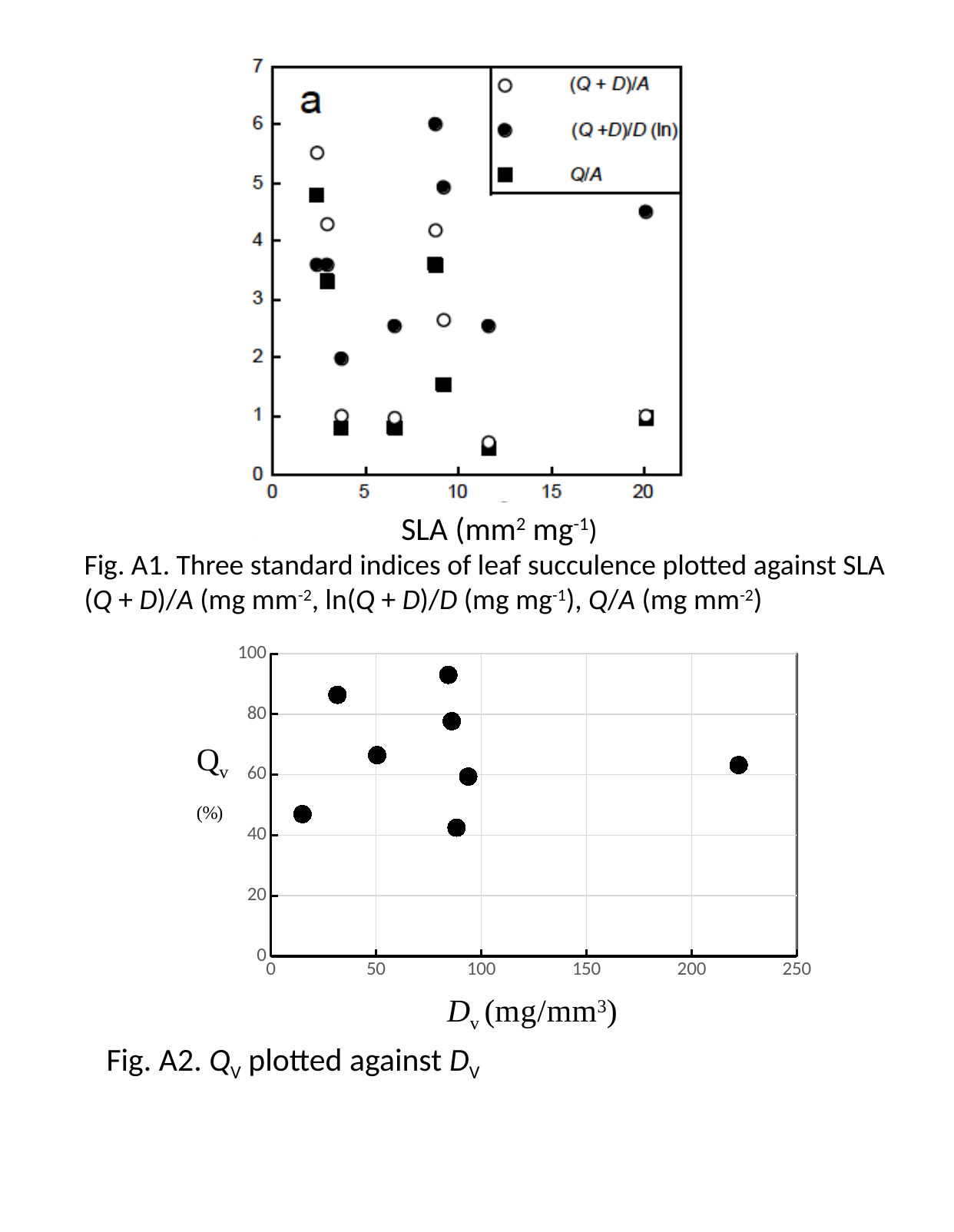

SLA (mm2 mg-1)
Fig. A1. Three standard indices of leaf succulence plotted against SLA
(Q + D)/A (mg mm-2, ln(Q + D)/D (mg mg-1), Q/A (mg mm-2)
### Chart
| Category | |
|---|---|Fig. A2. QV plotted against DV
